# Improving Collaboration Between Youth Peer Support Workers and Non-peer Colleagues in Child and Adolescent Mental Health Services

**DOI:** 10.1007/s10488-023-01283-w

**Published:** 2023-06-19

**Authors:** Carolijn R. M. de Beer, Lieke van Domburgh, Robert R. J. M. Vermeiren, Martin de Vreugd, Laura A. Nooteboom

**Affiliations:** 1 LUMC Curium - Department of Child and Adolescent Psychiatry, Leiden University Medical Center, Post Box 15, 2300 AA Leiden, The Netherlands; 2 Department of Child and Adolescent Psychiatry & Psychosocial Care, Amsterdam University Medical Center, Amsterdam, The Netherlands; 3 iHUB, Rotterdam, The Netherlands

**Keywords:** Youth, Lived experience workforce, Youth peer support workers, Clinicians, Child and adolescent mental health services, Collaboration

## Abstract

The involvement of youth peer workers (YPSWs) in child and adolescent mental health services (CAMHS) stimulates hope, destigmatization, and more culturally and developmentally appropriate support. Nevertheless, the collaboration between YPSWs and non-peer colleagues remains challenging, as it requires services to embed a new type of expert into practice. To stimulate the involvement of YPSWs in practice, this study reports on 27 semi-structured interviews with YPSWs and non-peer colleagues to provide insight into the barriers and facilitators in the collaboration process. The study took place in the Netherlands. A total of 10 interviews with YPSWs, and 17 interviews with non-peer colleagues in different healthcare occupations in CAMHS were conducted. Overall, the participants perceived relatively more barriers compared to facilitators in the collaboration process. Barriers to operate efficiently with YPSWs in multidisciplinary teams included: condescending attitudes and professional stigma towards YPSWs; concerns for YPSW boundaries; bureaucratic and clinical language usage by non-peer colleagues; conflicts due to different sets of expertise; and, lack of role clarity and guidelines for YPSWs. To improve the partnership between YPSWs and non-peer colleagues, participants described the importance of supervision and monitoring of YPSW activities. Moreover, participants also stressed the need for clear guidelines, and introduction and evaluation sessions to facilitate the collaboration process. While YPSWs seem to be an asset to CAMHS, there are a number of barriers to overcome. To overcome these barriers, organizational commitment, supervision (especially from peer colleagues), flexibility by non-peer colleagues, training non-peer staff to support YPSWs, and consistent evaluation of the implementation of YPSWs in services is recommended.

**Supplementary Information:**

The online version contains supplementary material available at 10.1007/s10488-023-01283-w.

## Introduction

The youth peer support workforce is rapidly expanding to child and adolescent mental health services (Tisdale et al., 2021). Youth peer support workers (YPSWs) are commonly identified as young adults with lived experience of mental illness during childhood or adolescence who are trained to provide emotional, practical and social support to young people with comparable experiences (Gopalan et al., 2017; Tisdale et al., 2021; de Beer et al., 2022). Even though the youth peer support workforce is expanding, the collaboration between YPSWs and non-peer colleagues in different healthcare professions remains challenging, because it requires services to embed a new type of expert into practice (Mancini, 2018; Tisdale et al., 2021; Byrne et al., 2021).

It is necessary to gain insight in how the collaboration between YPSWs and non-peer colleagues can be improved, as YPSWs can be valuable to young people experiencing mental health difficulties (Gopalan et al., 2017; Hiller-Venegas et al., 2022; Beer et al., 2022). For instance, YPSWs are often close in age to young people using child and adolescent mental health services (CAMHS), allowing YPSWs to offer culturally and developmentally appropriate support (Gopalan et al., 2017; Hiller-Venegas et al., 2022). This is underlined in a focus group study with transition-aged youth in publicly funded mental health services. They favored YPSWs who are just slightly older, because these YPSWs frequently experienced mental illness during a similar developmental period, which enhanced relatability to the experiences of transition-aged youth (Hiller-Venegas et al., 2022). Moreover, a qualitative study by Simmons et al. (2020) describes YPSWs add value to young people with mental health difficulties. This is based on their ability to connect as a result of their non-clinical identity, lived experience, less formal demeanor, and less formal language usage (Simmons et al., 2020). Overall, research on YPSWs suggests they stimulate self-acceptance, reduce stigma, and offer young people perspective by showcasing recovery is possible (Gopalan et al., 2017; Hiller-Venegas et al., 2022; Kidd et al., 2019; Simmons et al., 2020).

While YPSWs can be beneficial to young people in treatment for mental illness; the expertise and authenticity brought by YPSWs disrupts the traditional ways of medical practice commonly promoted by non-peer colleagues with a medical or clinical background. Such practice focuses on the medical deficit model, whereby treatment is protocolized and a hierarchical structure exists that favors a clinical or medical background (Manchini, 2018; Gillard, 2019). YPSWs endorse person-directed and recovery-oriented practice; a practice that values lived experience, patient autonomy, and finding personal meaning and moving on after having lived through mental distress (Byrne et al., 2021; Gillard, 2019; Manchini, 2018 ). Research proposes that due to these differences in expertise and approach, non-peer colleagues and YPSWs struggle to collaborate. For instance, some non-peer colleagues lack understanding of the unique contributions YPSWs can make to practice, and also fail to understand the nature of the YPSW role, which can cause anxiety, worsen professional stigma, and increases paternalistic behaviors towards YPSWs (Lambert et al., 2014; Tisdale et al., 2021; Hopkins et al., 2021). Moreover, research stipulates there is limited insight into the behaviors and workplace culture that enable the collaboration between YPSWs and non-peer colleagues (Byrne et al., 2021).

In order for YPSWs to make valuable contributions and have a safe work environment in CAMHS, it is necessary to gain insight into the barriers and facilitators in the partnership between YPSWs and non-peer colleagues. Through semi-structured interviews with YPSWs and non-peer colleagues in different occupations, we aim to shed light on these barriers and facilitators. In order to stimulate a safe work environment for YPSWs, and to strengthen the implementation of YPSWs in practice.

## Method

This study reports on the findings of semi-structured interviews with YPSWs and non-peer colleagues. The study is conducted in accordance with the consolidated criteria for reporting quality research guidelines (COREQ) (Tong et al., 2007). The medical ethics review board of the Leiden University Medical Center judged the overall study and stated that the research is not subject to the Medical Research Involving Human Subject Act (non-WMO approval number: N21.092). The board also concluded the study complies with the Netherlands Code of Conduct for Research Integrity.

### Participants

#### Non-peer Colleagues

To be included in this study, non-peer colleagues had to work in CAMHS as a psychologist, psychiatrist, doctor, family therapist, sociotherapist, social worker, case manager, or as a youth mental health policy-maker. We defined CAMHS, as services that provided both inpatient, outpatient and community care to young people between the ages of 12–21 with (severe) mental health problems. Recruitment took place through purposeful sampling at three CAMHS in the Netherlands: Leiden University Medical Center Department of Child and Adolescent Psychiatry (LUMC Curium), iHUB, and Pluryn. While LUMC Curium is a child and adolescent psychiatric treatment facility; iHUB and Pluryn are services that offer specialist care, education, and (housing) support for youth with disability, complex behavioral and psychological needs. We approached team managers and personal contacts within these services that knew potential participants (snowballing) and asked them to spread our recruitment letter. Potential participants either asked their contact person to send us their details; or contacted the first author (CB) directly through emailing the address provided at the bottom of the recruitment letter. The first author (CB) provided the participants with information on the study and asked the participants to sign an informed consent digitally prior to application of interview. None of participants withdrew consent after application of the interview.

#### Youth Peer Support Workers

To be included in this study, YPSWs had to have lived experience of mental illness and recovery during childhood or adolescence. In addition, to ensure YPSWs were able to share work related experiences, they had to employ this lived experience as an advocate, advisor, coach, mentor, buddy or counselor to influence child and adolescent mental health policy; teach and inform others about child and adolescent mental illness; and/ or, support young people in recovery from mental illness. Recruitment of YPSWs took place through purposeful snowball sampling at two locations: the National Youth Council and Experienced Experts (ExpEx). The Dutch National Youth Council is a panel consisting of young people who use their lived experience of mental illness to improve care and society for other young people with mental illness. ExpEx is a Dutch organization that trains and deploys YPSWs to support young people with mental illness and/or to advise policy makers and mental health services. We reached potential candidates through spreading the recruitment message via our personal contacts and contacts they knew (snowballing) within these organizations. Interested candidates either asked their contact person to send us their details; or contacted the first author (CB) directly through emailing the address provided at the bottom of the recruitment letter. The first author (CB) e-mailed potential candidates a letter with information on the study; planned a date for the interview; and asked candidates to sign and mail an informed consent digitally prior to application of interview. None of participants withdrew consent after application of the interview.

### Data Collection

To gain insight into the barriers and facilitators in the partnership between YPSWs and non-peer colleagues, semi-structured interviews were conducted. Two topic lists, one for YPSWs and one for non-peer colleagues, with open-ended questions were developed in reflective meetings with the authors of this study. Moreover, topics for the topic list were first formulated during two focus group consultation with YPSWs from the National Youth Council. The topic list for YPSWs was pilot tested on a YPSW (third author of this study). A broad range of topics on youth peer support were covered during the interviews, however, for the purpose of this study we focused on the following topics: barriers and facilitators for involving YPSWs; added value of YPSWs; and barriers and facilitators in partnership between YPSWs and non-peer colleagues. The interviews were conducted in Dutch, thus, the quotes presented in the "Results" section are translated and might therefore be subject to translator bias. Please see appendix B for the translated topic lists used for this study.

### Procedure and Setting

The interviews took place online through Microsoft Teams shortly after recruitment (recruitment dates: January 2022 – April 2022) between February 2022 and April 2022.The interviews lasted between 28 and 64 min and participants did not receive stipends. Interviews with YPSWs were conducted by the first author in collaboration with a YPSW (third author) and a student assistant from the Leiden University Medical Center. The YPSW was not present during interviews with non-peer colleagues to ensure non-peer colleagues felt free to express eventual concerns regarding youth peer support work. The interviews were recorded and transcribed verbatim. Field notes were obtained by the student assistant during the interviews. The authors (including the main interviewer) had previously conducted a systematic review on youth peer support, resulting in a positive-critical attitude towards implementing YPSWs (e.g. the authors see the added value of YPSWs, but understand there are numerous barriers to overcome in the implementation process). For most of the participants, except one YPSW and three non-peer colleagues, the interview was first time they met with the researchers.

### Analysis

The interview transcripts were imported to Atlas.Ti version 9, a qualitative data analysis software for labeling and organizing textual data. We applied the six steps for thematic analysis of qualitative data to guide the analysis process (Kiger & Varpio, 2020; Braun & Clarke, 2006). The steps included: (1) becoming familiar with the data, (2) generating initial codes, (3) grouping codes to generate themes, (4) reviewing and reflecting on themes, (5) defining, enhancing, and naming themes, and (6) locating exemplars and writing up results (Braun & Clarke, 2006). Since this was an exploratory study, we chose to conduct a thematic analysis as it allowed us to interpret, organize, analyze and describe the interview data in a coherent manner (Braun & Clarke, 2006). We further enhanced the thematic analysis by conducting a content analysis to quantify the frequency of given barriers and facilitators (Morgan, 1993). First, by transcribing and actively (re-)reading the interview transcripts we familiarized ourselves with the data. Subsequently, we generated initial codes, both inductive and deductive coding strategies were applied. The deductive coding approach was directed by developing a codebook based on the topic lists, this allowed for structure in the data and enabled us to answer the research question (see appendix A). The deductive coding was further enhanced by inductively adding codes that arose from the open coding of the interview transcripts to ensure no relevant data was missed. Two researchers, the first author and a student assistant coded the transcripts independently and discussed each transcript to resolve eventual differences. Then, axial coding took place by grouping together similar codes and by generating analytical themes. During reflective team meetings all authors of this study discussed the meaning and interpretation of the themes to further enhance and define a description of the themes. The final step entailed writing up the final analysis and description of data in the "Results"section below (Kiger & Varpio, 2020).

## Results

### Demographics

A total of 17 non-peer colleagues and 10 YPSWs were interviewed. Of the non-peer colleagues, 1 worked for Pluryn, 4 worked for iHUB, and 12 worked for LUMC Curium. Of the included non-peer colleagues, 16 had experience of working with YPSWs. The professional who did not have previous experience of working with YPSWs, did have experience with colleagues that used experiential expertise in practice. Of the YPSWs, a total of 5 YPSWs worked for the National Youth Council and 6 YPSWs worked for ExpEx (note: some YPSWs worked for both the National Youth Council and ExpEx). Moreover, next to working for the National Youth Council and/ or ExpEx, numerous YPSWs also worked for different organizations, including: child and adolescent psychiatry and specialist care services (n = 5); mental health foundations and charities (n = 4); and, training and education initiatives (n = 4). All of the included YPSWs previously participated in at least one training on youth peer support work. Although the type of training for YPSWs participating in these interviews varied, during interviews YPSWs described that that the following themes were addressed during the training(s): storytelling, active listening, (personal) boundaries, personal qualities and pitfalls, effective communication, recovery and empowerment, sharing lived experience safely and valuably, and supporting others. See Table 1 for an overview of the demographic characteristics of the non-peer colleagues and YPSWs.

**Table 1:** Demographic characteristics non-peer colleagues and YPSWs

Variable	
Non-peer colleagues	
Gender	
Male	6 (35.9%)
Female	11 (64.7%)
Non-binary	0 (0%)
Age in years	
Mean age in years	37.7
Age range in years (SD)	21–63 (11.48)
Occupation	
Psychiatrist [n(%)]	1 (5.9%)
Clinical psychologist [n(%)]	1 (5.9%)
Developmental psychologist [n(%)]	2 (11.8%)
Family therapist	1(5.9%)
Policy advisor youth care [n(%)]	1 (5.9%)
Manager social therapists [n(%)]	1 (5.9%)
Social therapist [n(%)]	5 (29.4%)
Social therapist in training [n(%)]	2 (11.8%)
Social worker[n(%)]	1 (5.9%)
Case manager and visual arts teacher	1 (5.9%)
Coach and trainer healthcare non-peer colleagues [n(%)]	1 (5.9%)
Youth peer support workers	
Gender	
Male [n(%)]	0 (0%)
Female [n(%)]	10 (100%)
Non-binary [n(%)]	0 (0%)
Age in years	
Mean age in years	24.1
Age range in years (SD)	21–32 (3.47)
Roles of YPSWs in practice^1^	
Engagement & emotional support (counseling, supporting & mentoring)	8
Advocacy	8
Research	3
Education (teaching and training)	6

^1^Number does not add up to 10 because a number of YPSWs took on more than one role

### Barriers and Facilitators in the Collaboration Between Non-peer Colleagues and YPSWs

**Fig. 1:**
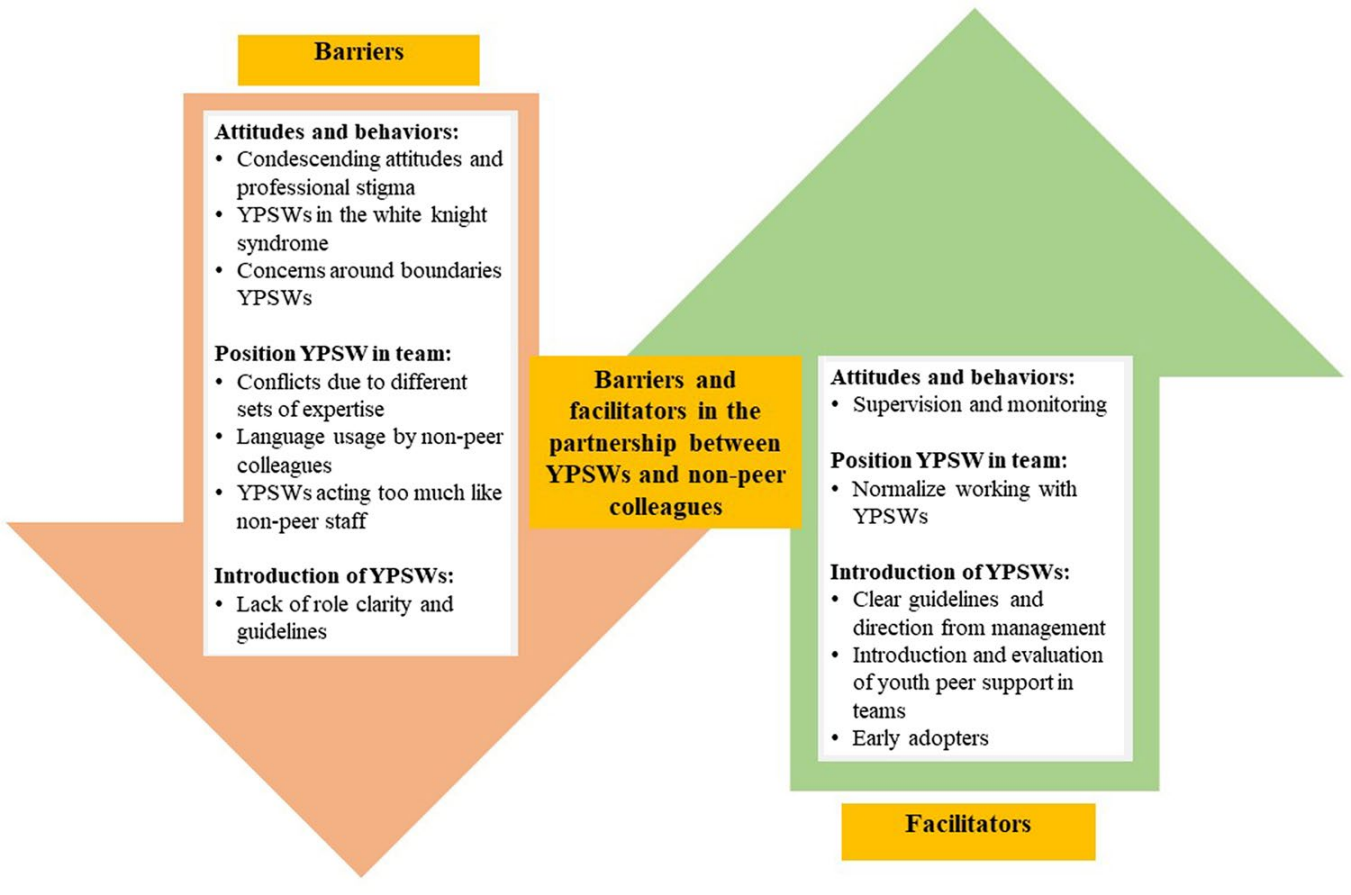
Barriers and facilitators within the collaboration process of YPSWs and non-peer colleagues

Overall non-peer colleagues and YPSWs described several facilitators and barriers they witnessed when working together. The findings from our thematic analysis are divided into three themes with barriers and facilitators for collaboration between non-peer colleagues and YPSWs. See Table 2; Fig. 1 for an overview of these themes and associated barriers and facilitators. The section below explores these three themes in more depth.

**Table 2:** Overview of themes and the associated barriers and facilitators

Theme	Barriers and facilitators	Barrier/ facilitator described by non-peer colleagues at least once during the interview (n)	Barrier/ facilitator described by YPSW at least once during the interview (n)
Attitudes and behaviors non-peer colleagues and YPSWs	Condescending attitudes and professional stigma of non-peer colleagues towards YPSWs (Barrier)	7 (41.2%)	8 (80%)
	YPSWs and the white knight syndrome (Barrier)	8 (47.1%)	5 (50%)
	Concerns around boundaries of YPSWs (Barrier)	15 (88.2%)	6 (60%)
	Supervision and monitoring of YPSWs (Facilitator)	8 (47.1%)	7 (70%)
Position of YPSWs in treatment teams	Conflicts due to different fields of expertise (Barrier)	9 (52.9%)	8 (80%)
	Clinical and bureaucratic language usage (Barrier)	2 (11.8%)	7 (70%)
	YPSWs acting too much like non-peer staff (Barrier)	8 (47.1%)	6 (60%)
	Normalize having YPSWs in your team (Facilitator)	3 (17.7%)	6 (60%)
Introducing YPSWs	Lack of job clarity and need for guidelines (Barrier)	15 (88.2%)	5 (60%)
	Clear guidelines and direction from management (facilitator)	12 (70.6%)	5 (50%)
	Introducing and evaluating the implementation of YPSWs (Facilitator)	14 (82.4%)	6 (60%)
	Early adopters (Facilitator)	10 (58.8%)	6 (60%)

#### Theme 1: Attitudes and Behaviors of Non-peer Colleagues and YPSWs

Both non-peer colleagues and YPSWs described different attitudes and behaviors towards one another that impacted the collaboration process. In this section the attitudes and behaviors that either improved or hindered the collaboration process between non-peer colleagues and YPSWs are described.

#### Condescending Attitudes and Professional Stigma of Non-peer Colleagues Towards YPSWs

A commonly mentioned barrier by some non-peer colleagues and most YPSWs in policy, treatment, and research were recurring condescending attitudes and professional stigma towards YPSWs by non-peer colleagues. YPSWs emphasized some non-peer colleagues were too cautious with them because of their openness and histories with mental illness. YPSWs described it felt demeaning when non-peer colleagues gave them excessive praise or overemphasized the importance of having YPSWs in meetings. In addition, some non-peer colleagues described some coworkers ignored the ideas and efforts made by YPSWs in team meetings.

“I don’t like being patronized by my colleagues. Compared to other colleagues, I notice they more often ask me if I’m doing okay and if the workload isn’t too demanding for me… In such cases I think ‘dude, who knows, maybe three other colleagues are super depressed and still come to work.’ Sometimes it kind of makes me feel like a misfit”.

YPSW 7

#### YPSWs and the White Knight Syndrome

In addition, a frequently described barrier by YPSWs and non-peer colleagues included the attitudes some YPSWs have towards fixing everything ‘broken’ within CAMHS. One professional described he felt as if he was constantly critiqued by YPSWs in treatment-settings for how he engaged and treated young people. Moreover, some non-peer colleagues who worked with YPSWs in policy and treatment settings described it was hard for them to work with YPSWs, as they felt the motivation of YPSWs were colored by their past adverse experiences within the youth mental health system.

“I certainly think having YPSWs can be an asset, however, my practical experience with YPSWs is they are often too rigid in holding on to their beliefs. It is almost as if they feel this job is their true calling. This compromises their professionalism.”

Non-peer colleague 1

#### Concerns Around the Boundaries of YPSWs

Another area of concern described by non-peer colleagues were the lack of clear boundaries some YPSWs in the engagement and emotional support role had when they engaged with young people. While non-peer colleagues argued that a strength of working with YPSWs is their ability to form authentic relationships based on equality and trust. Non-peer colleagues also feared that this could lead to young people overstepping the boundaries of YPSWs. Non-peer colleagues described that this made them more resistant to include YPSWs in practice.

“Sometimes I hear young people ask questions to our YPSW that are really crossing the line. Young people sometimes just seek sensation and commotion, instead of wanting to hear about the experiences of the YPSW.”

Non-peer colleague 15

#### Supervision and Monitoring

Both non-peer colleagues and YPSWs felt that having regular supervision from a committed member of staff, with status and connections in the workplace, facilitated YPSWs in setting professional boundaries and managing the workload. Participants described supervision to be beneficial as it helped YPSWs in all roles to reflect on the young people they supported and difficult interactions with non-peer colleagues. Moreover, some participants felt it was valuable when the supervisor also had an active role in monitoring the activities and implementation process of YPSWs. When required supervisors could connect YPSWs to colleagues to ensure YPSWs become well established within the workplace.

#### Theme 2: YPSW Position Within Multidisciplinary Teams

This second theme explores the barriers and facilitators for creating a valuable position for YPSWs within multidisciplinary teams in treatment, policy and research.

#### Conflicts Due to Different Fields of Expertise

The expertise of YPSWs is based on lived experience and training in core principles of peer support, whereas the expertise of non-peer colleagues is often theory driven and frequently based on clinical practice, policy or research. Even though these different sets of expertise of YPSWs and non-peer colleagues can complement one another, they can also form a barrier when they clash. For example, YPSWs described they often had to protect their stance and viewpoints in meetings with colleagues, making it hard for YPSWs to establish themselves within teams. While, non-peer colleagues felt that the experiential expertise of YPSWs was too personal to the YPSW, limiting its generalizability.

#### Language Usage

In addition, YPSWs in research, advocacy, and treatment expressed they had difficulties finding their place within teams as non-peer colleagues frequently used clinical and bureaucratic language they did not understand. Both YPSWs and non-peer colleagues have differing backgrounds and sets of expertise, and for them to collaborate it is important to communicate in terms both parties understand.

“I think YPSWs are not always approached as equals when invited to the table. When collaborating with YPSWs, some non-peer colleagues tend to only use clinical language. YPSWs are very capable, but when non-peer colleagues use a certain type of language they cannot participate.”

YPSW 3

#### Acting too Much Like Non-peer Staff

In search for a valuable position within multidisciplinary teams in clinical settings and policy, some YPSWs attempted to fit in by mirroring other non-peer colleagues. This was described as a barrier by both non-peer colleagues and YPSWs.

“Now that I am studying to become a psychologist, I’m kind of drilled to think like a psychologist. I notice that in my role as YPSW, I sometimes approach others through the lens of a psychologist. I am aware this is a risk, and I try to find a balance between both roles.”

YPSW 5

#### YPSW 5 Normalize Having YPSWs in a Team

Finally, participants noted that to facilitate the collaboration process and to create a position for YPSWs within multidisciplinary teams, it is important for non-peer colleagues to normalize having YPSWs in teams. Participants described that some non-peer colleagues felt the need to plan formal events in which YPSWs could participate. In doing so, the involvement of YPSWs becomes a unique event, and YPSWs are alienated.

#### Theme 3: Introducing YPSWs

This final theme explores barriers and facilitators that either hindered or enabled the introduction of YPSWs within multidisciplinary teams.

#### Job Clarity and Need for Guidelines

A frequently mentioned barrier by both YPSWs in all roles and non-peer colleagues, was the lack of job clarity and existing guidelines for YPSWs in practice. Many non-peer colleagues described that for them, it was often not clear how the skills and expertise of YPSWs could be applied to practice. This hindered non-peer colleagues in their partnership with YPSWs. They described that guidelines could facilitate non-peer colleagues to support YPSWs starting out. YPSWs also described they needed more direction from management and colleagues to function well.

“When I started 3 years ago I didn’t know what I could do. I had a work contract for 20 hour each week, but I didn’t know how to fill those hours. The start was very messy, which was hard for me, as I am a person that needs a lot of direction to function well. Eventually I decided to organize my own work and to approach co-workers to ask if I could help them. I slowly grew in my role as YPSW. Now our service has a whole team consisting of YPSWs.”

YPSW 1

#### Introducing and Evaluating the Implementation of YPSWs

As a facilitator many non-peer colleagues and YPSWs (employed to see young people face-to-face and in policy) described that introducing YPSWs during team meetings was helpful. Non-peer colleagues described that preparation sessions allowed them to ask questions to YPSWs and to make setting-specific arrangements for YPSWs in practice (e.g. brainstorming themes and projects in which the inclusion of lived experience is vital). Once introduced, evaluation sessions between the YPSW and specifically appointed enthusiastic coworkers was helpful to continue collaboration with YPSWs; and to resolve potential problems faced by teams starting out with YPSWs.

“Within our team everyone has different expectations for YPSWs. As a team, it is important to sit with the YPSW to make arrangements and set expectations. It is also important that everyone is open about what they feel and think of youth peer support. Some people enjoy having YPSWs around, while others do not see the added value of a YPSW and may tell the YPSW to leave. Setting expectations and arrangements for the YPSW as a team, including the YPSW, and frequently evaluating these with the YPSW, allows for everyone to be on the same page. So don’t brush it off, take the YPSW seriously”

Non-peer colleague 16

#### Early Adopters

Finally, a total of ten non-peer colleagues and six YPSWs (employed in policy, research, education, engagement and emotional support roles) described that having non-peer colleagues approach peer support with enthusiasm was helpful in the implementation process of YPSWs. Non-peer colleagues described having colleagues act as early adopters for youth peer support provided them with a space to ask questions and address concerns about youth peer support. Eventually, building upon the existing enthusiasm of early adopters will allow for a ripple effect within the organization, facilitating the implementation and pursuit of services by YPSWs.

“We need early adopters, I am not sure if you have heard of that term before? You start with a small group of people who are really enthusiastic about peer support, and eventually this group will naturally continue to grow… It’s a way to build upon and increase the existing enthusiasm.”

Non-peer colleague 8

## Discussion

This study explored the barriers and facilitators for beneficial collaboration between YPSWs and non-peer colleagues. Overall the participants perceived relatively more barriers compared to facilitators. This suggests that the implementation of YPSWs in practice is a complicated process requiring careful planning, organizational commitment and frequent monitoring. Below we examine the described barriers and facilitators to provide recommendations to strengthen the partnership between non-peer colleagues and YPSWs in practice.

As can be concluded from the interviews, due to professional stigma and excessive praise, YPSWs felt underacknowledged and marginalized. This finding aligns with previous research, the perceived vulnerability of YPSWs can result in non-peer colleagues lacking confidence in YPSWs, and YPSWs lacking confidence in themselves (Tisdale et al., 2021; Delman and Klodnick, 2017; de Beer et al., 2022). YPSWs have a dedicated professional role based on lived experience and training, but are treated differently (e.g. treated with caution by colleagues) and stigmatized for having lived experience in the first place. Moreover, seeing that YPSWs are commonly young adults with limited work experience, the perceived vulnerability of YPSWs by non-peer colleagues is further increased. Studies suggest YPSWs need time, professional development opportunities, and experience to grow confident in their new function (Simmons et al., 2020; de Beer et al., 2022). Therefore, it is likely, that as YPSWs gain more experience and confidence in their role with time, professional stigma and excessive praise by non-peer colleagues towards YPSWs will decrease. In order to implement YPSWs successfully, employers must provide YPSWs with training opportunities to assist YPSWs in further developing their skills. Moreover, non-peer colleagues must be aware that it can take time for YPSWs to take on their new role, so that they can manage their expectations. In addition, it is crucial that non-peer colleagues actively involve YPSWs, to provide them with ample practical opportunities to gain experience and hone their skills. YPSWs should not be excluded, after all their (prior) vulnerabilities are the very reason they are hired in the first place.

Another barrier in the collaboration process included concerns by non-peer colleagues on YPSWs overstepping boundaries when supporting young people. This concern is not surprising as previous research underlines the seemingly informal nature of the relationships between YPSWs and service users; a relationship commonly described as ‘approximating friendship’ (Tisdale et al., 2021; Halsall et al., 2021). While, peer relationships between YPSWs and young people seem to approach friendship, it is not the same. YPSWs are employed, have been trained, and use a risk protocol to assess if young people are a danger to themselves or others (Scott, 2011). However, in line with the results and previous studies, we do stress the importance that services provide supervision to YPSWs (Tisdale et al., 2021; Delman & Klodnick, 2017; Mancini, 2017). Supervision is deemed crucial to manage boundaries and improve collaboration between YPSWs and non-peer colleagues (Tisdale et al., 2021; Delman & Klodnick, 2017). Moreover, supervision can enable YPSWs to navigate pressure of acceptance by colleagues when they feel undervalued (Tisdale et al., 2021). While our results recommend supervision from a committed member of staff to be valuable, research indicates lived experience supervision from peer colleagues is superior to reduce isolation and the alienation YPSWs can experience when they are the only YPSW in a team (Mancini, 2018).

Overall, perceived barriers, such as ‘lack of role clarity for YPSWs’, ‘clinical and bureaucratic language usage by non-peer colleagues’, and ‘conflicts when collaborating due to different sets of expertise’, can be linked back to the authentic and diverse nature of YPSWs. Unlike the expertise of non-peer colleagues, the expertise provided by YPSWs is unique to each YPSW and difficult to replicate (Gillard, 2019). In an attempt to research and feel comfortable with YPSWs in clinical settings, we tend to feel the need to create guidelines and to pinpoint replicable elements of youth peer support work. However, in doing so youth peer work is restricted (Byrne et al., 2021; Gillard, 2019; Mancini, 2018). This is also evident from the quote of YPSW 5 in the "Results" section, which describes the risk for being absorbed by approaches more commonly resembling the work of non-peer colleagues, such as psychologists. Although it can feel comfortable and reassuring for non-peer colleagues seeing YPSWs in similar roles, it erodes the fundamental principles of youth peer support being done. Instead, to collaborate with YPSWs successfully and to avoid tokenism, it is important to advocate, normalize, and consider the setting the YPSW works in. In addition, the implementation process of YPSWs needs to be evaluated in multidisciplinary teams (de Beer et al., 2022; Lambert et al., 2014; Hopkins et al., 2021). In agreement with Hopkins et al. (2021), we suggest that non-peer colleagues who have been working with the medical deficit model should adapt their language and viewpoints when collaborating with YPSWs, even when it feels uncomfortable. Having some non-peer colleagues act as early adopters, can be helpful for non-peer colleagues who still have concerns for youth peer support. Moreover, prior to introducing YPSWs it is important to inform non-peer staff on the roles YPSWs can take on, and to provide them with training on how they can best support YPSWs to bring their expertise to practice (Lambert et al., 2014). Role confusion can give rise to numerous responses of non-peer colleagues, from disdain (e.g., ignoring efforts made by YPSWS) to overly enthusiastic praise. When youth peer worker roles are understood, YPSWs, the young people they work with, services, and the broader child adolescent mental health system all benefit.

### Strengths and Limitations

The use of semi-structured interviews allowed for in-depth exploration of the barriers and facilitators experienced by YPSWs and non-peer colleagues in CAMHS. By employing a qualitative research design, we enhanced the exploratory nature of this study and ensured potential context depended nuances were accounted for. The involvement of numerous non-peer colleagues in a wide range of disciplines allowed for a diverse range of perspectives from non-peer colleagues on youth peer support work. However, it should be noted that while a large number of healthcare professions are represented by the non-peer colleagues participating in the interviews, there are few participants in each group. Moreover, through collaborating with a YPSW during the development and application of the interviews for YPSWs, we improved rapport with the YPSWs that participated in the interviews. This allowed for deep insight in the experiences of YPSWs.

Nevertheless, this study should be interpreted in light of a few limitations. First, in the recruitment process a purposive and convenience sampling approach was taken. This limits our findings to a selective group of individuals who may have strong opinions on youth peer support work. We attempted to increase generalizability through approaching YPSWs and non-peer colleagues in a number of organizations in the Netherlands (iHUB, Pluryn, LUMC Curium, ExpEx and the National Youth Council). However, of the 17 non-peer colleagues, a total of 12 worked for LUMC Curium. Thus, the perspectives of non-peer colleagues are mainly a reflection of those employed at LUMC Curium. Another limitation included the overrepresentation of female YPSWs; indicating the perceptions of YPSWs might be subject to gender bias. Besides, while this study consults both non-peer colleagues and YPSWs, future studies should also approach young people receiving treatment to gain insight into how YPSWs can be involved next to non-peer colleagues to be beneficial for young people in CAMHS. In addition, this study is a reflection of the experiences of non-peer colleagues and YPSWs, observational research, such as case studies, should be undertaken to observe (unconscious) behavior influencing the partnership between YPSWs and non-peer colleagues. Finally, considering that in our study YPSWs worked in different and often multiple settings, we did not differentiate whether YPSWs worked alone or in teams with other peer colleagues. However, since working alone can make YPSW more vulnerable to stigma (Mancini, 2018), we recommend future studies to deepen our insight in potential differences between YPSWs working alone or in teams with other YPSWs.

## Conclusion

Overall, this study allowed for valuable insight into the barriers and facilitators for improved collaboration between YPSWs and non-peer colleagues. While YPSWs seem to be an asset to CAMHS, the involvement of YPSWs can be challenging and requires both non-peer colleagues and YPSWs to overcome numerous barriers. Most of these barriers in the partnership between non-peer colleagues and YPSWs can be linked to the (perceived) vulnerability, authenticity and openness YPSWs bring to practice. To overcome these barriers, flexibility and understanding by non-peer colleagues, supervision (specifically from peer staff), organizational commitment, staff preparation, and consistent evaluation of the involvement of YPSWs in practice is required.

## Supplementary Information

Below is the link to the electronic supplementary material.

Supplementary material 1 (DOCX 27.7 kb)

Supplementary material 2 (DOCX 19.1 kb)

## Data Availability

The datasets generated and analyzed during the current study are available from the corresponding author on reasonable request.

## Abbreviations

YPSWs: Youth peer support workers
CAMHS: Child and adolescent mental health services
