# Supplementary material for: Improving Collaboration Between Youth Peer Support Workers and Non-peer Colleagues in Child and Adolescent Mental Health Services
Source: Adm Policy Ment Health. 2023 Jun 19;50(5):824–33. doi: 10.1007/s10488-023-01283-w (PMC10359199; doi:10.1007/s10488-023-01283-w)
Supplement: Supplementary file 1 — Supplementary material 1 (DOCX 27.7 kb) [file 10488_2023_1283_MOESM1_ESM.docx]

**Appendix A:** codebook with predefined codes and codes added after open coding of the interviews

| Code | Description | Related themes | Related barrier/ facilitator |
| --- | --- | --- | --- |
| Predefined codes (topic list) | | | |
| YPSW factors facilitating collaboration | A global code to collect factors that improved collaboration with YPSWs. | Attitudes and behaviors mental health professionals and YPSWs | Supervision |
|  |  | Introducing YPSWs | Introducing and evaluating the implementation of YPSWs |
| YPSW factors hindering collaboration | A global code to collect factors that hindered collaboration with YPSWs. | Attitudes and behaviors mental health professionals and YPSWs | White knight syndrome |
|  |  |  | Boundaries YPSWs |
|  |  | YPSW position within treatment team | Different fields of Expertise |
|  |  |  | Acting too much like a healthcare professional |
|  |  | Introducing YPSWs | (Lack of) role clarity and guidelines YPSWs |
| Factors facilitating collaboration with professionals | A global code to collect factors that improved collaboration with professionals. | Attitudes and behaviors mental health professionals and YPSWs | Supervision |
|  |  | YPSW position within treatment team | Normalize having YPSWs in teams |
|  |  | Introducing YPSWs | Introducing and evaluating the implementation of YPSWs |
|  |  |  | Early adopters |
| Factors hindering collaboration with professionals | A global code to collect factors that hindered collaboration with professionals. | Attitudes and behaviors mental health professionals and YPSWs | Condescending attitudes and behaviors professionals |
|  |  | YPSW position within treatment tram | Different fields of expertise |
|  |  |  | Clinical language usage |
| Organizational needs for YPSW | Needs within an organization to facilitate youth peer support. | Attitudes and behaviors mental health professionals and YPSWs | Supervision |
|  |  | YPSW position within treatment teams | Normalize having YPSWs |
|  |  | Introducing YPSWs | Early adopters |
| Support needs YPSWs | Required support from colleagues and organizations to work as a YPSWs. | Attitudes and behaviors mental health professionals and YPSWs | Supervision |
|  |  | YPSW position within treatment teams | Normalize having YPSWs |
|  |  | Introducing YPSWs | Early adopters |
| Open coding | | | |
| Belittling of YPSWs | At times YPSWs felt belittled by professionals. | Attitudes and behaviors mental health professionals and YPSWs | Condescending attitudes and behaviors professionals |
| Vulnerability YPSWs | Perceived vulnerability of YPSWs impacted approach taken by professionals towards YPSWs. | Attitudes and behaviors mental health professionals and YPSWs | Condescending attitudes and behaviors professionals |
| Fear of triggering YPSWs | Some professionals fear triggering/ relapse within YPSWs. | Attitudes and behaviors mental health professionals and YPSWs | Condescending attitudes and behaviors professionals |
| Motivation colored by adverse experiences | Past negative experiences impacted the ways YPSWs approached their work. | Attitudes and behaviors mental health professionals and YPSWs | White knight syndrome |
|  |  | YPSW position within treatment teams | Different fields of expertise |
| YPSWs as savior | Strong desires within YPSWs to improve services. This desire is based on past negative experiences with services. | Attitudes and behaviors mental health professionals and YPSWs | White knight syndrome |
| Rigidity in beliefs | Professionals described YPSWs were at times too rigid in holding on to their beliefs. This is related to desire to improve mental health services based on personal negative experiences with services. | Attitudes and behaviors mental health professionals and YPSWs | White knight syndrome |
|  |  | YPSW position within treatment teams | Different fields of expertise |
| Concerns boundaries | The nature of relationships (openness, authenticity etc.) between YPSWs and young people, can make it hard to manage boundaries. Professionals feared young people overstepping boundaries of YPSWs. | Attitudes and behaviors mental health professionals and YPSWs | Boundaries YPSWs |
| (lack of) professionalism YPSWs | Relationships of YPSWs with young people ‘approaches friendship’. Moreover, YPSWs were at times seen as service users. | Attitudes and behaviors mental health professionals and YPSWs | Boundaries YPSWs |
| Supervision | Supervisions allows YPSWs to manage boundaries and evaluate relationships with staff and young people. Also helps YPSWs to take steps back when needed, and practice appropriate self-care. | Attitudes and behaviors mental health professionals and YPSWs | Boundaries YPSWs |
|  |  |  | Supervision |
| Clinical practice vs. lived experiences | Different fields of expertise resulting in opposing viewpoints and misunderstanding. | YPSW position within treatment teams | Different fields of expertise |
|  |  |  | Clinical language usage |
| Protecting viewpoints | Professionals and YPSWs sometimes felt they had to protect their ideas, beliefs and roles. | YPSW position within treatment teams | Different fields of expertise |
| Misunderstanding | Misunderstanding between YPSWs and professionals based on lack of insights in each other’s background and expertise. | YPSW position within treatment teams | Different fields of expertise |
|  |  |  | Clinical language usage |
| Language usage | Hard for YPSWs to participate when clinical and bureaucratic language is used. | YPSW position within treatment teams | Clinical language usage |
| Finding ways to fit in | Flexible roles and lack of direction around roles, meant YPSW tried to fit in by mirroring professionals or finding gaps they could fill. | YPSW position within treatment teams | Acting too much like a healthcare professional |
| (Lack of) role clarity | Lack of guidelines and flexible nature youth peer support work, meant limited understanding in roles and tasks for YPSWs. Guidelines were helpful for both YPSWs and professionals. | YPSW position within treatment teams | Acting too much like a healthcare professional |
|  |  | Introducing YPSWs | (Lack of) role clarity and guidelines YPSWs |
| Missing understanding added value YPSWs | Professionals lacked understanding on the added value of YPSWs. | Introducing YPSWs | (Lack of) role clarity and guidelines YPSWs |
| Evaluation | Evaluation facilitated the implementation and pursuance of youth peer support work. | Introducing YPSWs | Introducing and evaluating the implementation of YPSWs |
| Normalization of YPSWs | YPSWs need to be involved frequently, and approached often to avoid tokenism. | YPSW position within treatment teams | Normalize having YPSWs |
| Isolation and alienation of YPSWs | YPSWs often sporadically involved in organizations. This results in alienation for YPSWs and tokenism. | YPSW position within treatment teams | Normalize having YPSWs |
| Preparation sessions | Introducing YPSWs and agreeing on plans for the tasks of YPSWs facilitated the implementation process. | Introducing YPSWs | Introducing and evaluating the implementation of YPSWs |
| Enthusiastic coworkers | Enthusiastic coworkers excited for youth peer support helped make space for YPSWs. | Introducing YPSWs | Early adopters |
| Early adopters | Having enthusiastic early adopters involved in the implementation of YPSWs, helped the involvement and pursuit of youth peer support. | Introducing YPSWs | Early adopters |
