# Supplementary material for: Improving Collaboration Between Youth Peer Support Workers and Non-peer Colleagues in Child and Adolescent Mental Health Services
Source: Adm Policy Ment Health. 2023 Jun 19;50(5):824–33. doi: 10.1007/s10488-023-01283-w (PMC10359199; doi:10.1007/s10488-023-01283-w)
Supplement: Supplementary file 2 — Supplementary material 2 (DOCX 19.1 kb) [file 10488_2023_1283_MOESM2_ESM.docx]

**Appendix B: Topic lists interviews**

***Topic list youth peer support workers***

| **Topic list for youth peer support workers** | **Example questions** |
| --- | --- |
| **Introduction** | Information regarding research project and process of the interview, informed consent, and voluntary nature of the interview. |
| **Motivation to participate and background information** | - Can you tell me more about why you decided to participate in the interview today? - How old are you? - Can you tell me more about your work as a youth peer support worker? What does a typical day entail? - Why did you choose to work as a youth peer support worker in the in youth-serving context? - Why do you think it is important to involve youth peer support workers in practice? |
| **Added value of youth peer support** | - Have you ever worked with a (youth) peer support worker yourself? Yes: How did you experience this? No: would this have been helpful for you?   - If applicable: how do you engage and connect with young people? - Are there areas in your work that are going well? Can you tell me more about this? - Do you ever get compliments from young people and/ or colleagues for the work you do? do? If so, what type of compliments? - How would you describe the added value of youth peer support? |
| **Roles and age youth peer support workers** | - What type of roles can youth peer support workers fulfill in the youth serving context? Do you have examples? - Is there a certain stage or age when you are no longer able to work as a youth peer support worker in youth-serving context? (including policy, research, education and face-to-face with young people). - When and for whom is it important and valuable to work with YPSWs? |
| **Training and skills youth peer support workers** | - What kind of education or training have you followed to work as a youth peer support worker? - Did you miss subjects/ training areas/ skills during your training as a youth peer support worker? For example: things that might have been relevant for your work today? - What type of skills are beneficial for your work as a youth peer support worker? - If you were asked to train or educate future youth peer support workers, what would you tell and/ or teach them? |
| **Recovery** | - How would you describe recovery? What entails recovery for you?   - According to your definition of recovery: When would you say you are ready to work as a youth peer support worker? Does this also depend on the type of role you are going to fulfill in the youth serving context? |
| **Guidance, supervision and support** | - What organizational requirements need to be in place to stimulate and facilitate the employment of youth peer support workers? - What type of guidance and support is important for you to work well? |
| **Communication and barriers and facilitators in partnership with colleagues** | - Have you experienced barriers in the partnership with non-peer colleagues? - Can you tell me more about things that are going well in contact/ partnership with (non-peer) colleagues? |
| **Facilitators and barriers during implementation and pursuit of youth peer support services** | - Would you like to tell us when you started as a youth peer support worker? How did you experience this? - Are there aspects within the youth serving context that can make it more difficult to introduce and pursue youth peer support services? - Are there things in your employment that have made it difficult for you to pursue your work? - Are there factors within organizations that can hinder your work as a youth peer support worker? |
| **Self-Disclosure** | - What is it like for you to share your own experiences with mental health difficulties and recovery with young people? - What is it like for you to share your own experiences with mental health difficulties and recovery with (non-peer) colleagues? - What do you think of clinicians who share openly about their own past with psychological complaints and adversities? - In your opinion: how does self-disclosure and personal sharing differ between youth peer support workers and non-peer colleagues (refers to clinicians)? |

***Topic list healthcare professionals***

| **Topic list for healthcare professionals** | **Example questions** |
| --- | --- |
| **Introduction** | Information regarding research project and process of the interview, informed consent, and voluntary nature of the interview. |
| **Motivation to participate and background information** | - Can you tell me more about why you decided to participate in the interview today? - How old are you? - Can you tell me more about your work? What does a typical day entail? - Can you tell me about your experience with youth peer support workers? |
| **Added value of youth peer support** | - How would you describe the added value of youth peer support? Do you have examples? |
| **Roles and age youth peer support workers** | - What type of roles can youth peer support workers fulfill in the youth serving context? Do you have examples? - Is there a certain stage or age when you are no longer able to work as a youth peer support worker in youth-serving context? (including policy, research, education and face-to-face with young people). - When and for whom is it important and valuable to work with YPSWs? |
| **Training and skills youth peer support workers** | - What type of skills are beneficial for youth peer support workers to have in the youth-serving context? |
| **Recovery** | - How would you describe recovery for YPSWs? What entails recovery for YPSWs you?   - According to your definition of recovery: When would you say a youth peer support worker is able to be employed as a youth peer support worker? Does this also depend on the type of role a youth peer support worker is going to fulfill in the youth serving context? |
| **Guidance, supervision and support** | - What organizational requirements need to be in place to stimulate and facilitate the employment of youth peer support workers? - What type of guidance and support is important for you to work well with youth peer support workers? What type of support is valuable for youth peer support workers? |
| **Communication and barriers and facilitators in partnership with colleagues** | - Have you experienced barriers in the partnership with peer support workers? - Can you tell me more about things that are going well in contact/ partnership with youth peer support workers? |
| **Facilitators and barriers during implementation and pursuit of youth peer support services** | - Are there aspects within the youth serving context that can make it more difficult to introduce and pursue youth peer support services? - Are there things in your employment/ setting that can make it difficult for you to include and work with youth peer support workers? - Are there factors within organizations that can hinder the pursuit and employment of peer support workers? |
| **Self-disclosure** | - Do you share your own experiences with mental health difficulties and recovery with young people? - Do you to share your own experiences with mental health difficulties and recovery with colleagues? - What do you think of non-peer colleagues who share openly about their own past with psychological complaints and adversities? - In your opinion: how does self-disclosure and personal sharing differ between youth peer support workers and non-peer colleagues? |
